# Supplementary material for: Chromone-Containing Allylmorpholines Influence Ion Channels in Lipid Membranes via Dipole Potential and Packing Stress
Source: Int J Mol Sci. 2022 Sep 30;23(19):11554. doi: 10.3390/ijms231911554 (PMC9570167; doi:10.3390/ijms231911554)
Supplement: Supplementary file 1 [file ijms-23-11554-s001.zip › ijms-1936985-supplementary.pdf]

Supplementary Material

1.1 Supplementary Figures

Supplementary Figure S1. Dependence of the decrease in the boundary potential of the membrane ($-\Delta\varphi_b$) on the concentration of chromone-containing allylmorpholines. The relation between the color of symbol and the compound is given on the figure legend. The membranes were composed of POPC and bathed in 0.1 M KCl at pH 7.4. V = 50 mV.

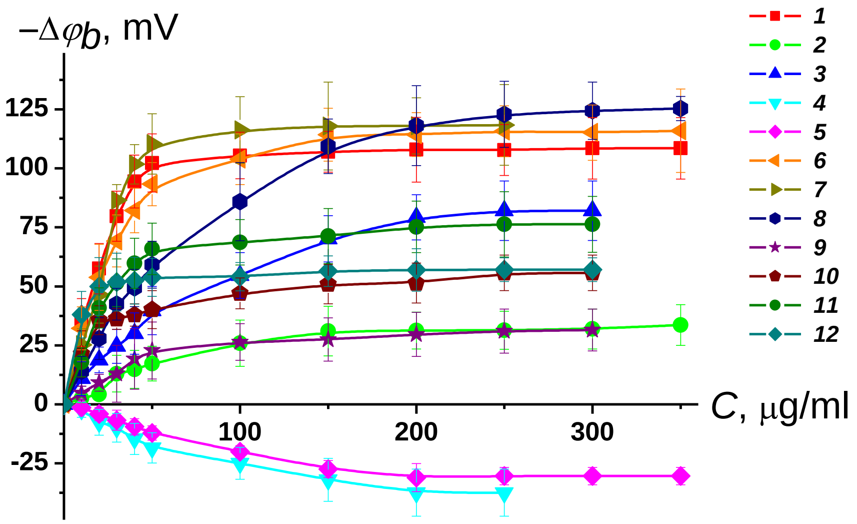

Supplementary Figure S2. Deconvolution analysis of the main transition peak of DPPC in the presence of 100 (*left panel*) and 250 μg/ml (*right panel*) of chromone-containing allylmorpholines. The parameters characterizing the individual components are summarized in Supplementary Table 2.

agent	*100 μg/ml*	*250 μg/ml*
1	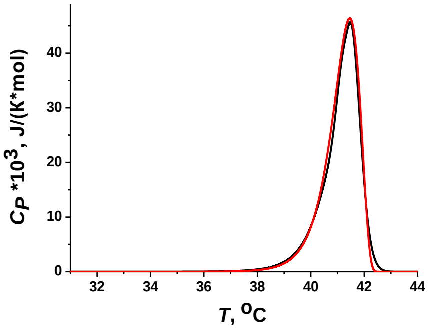	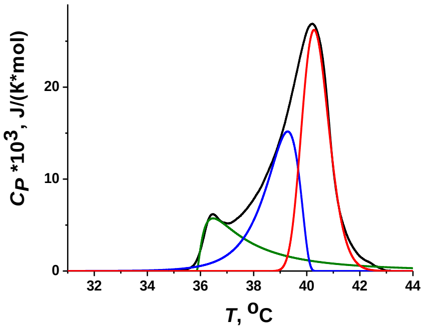

2

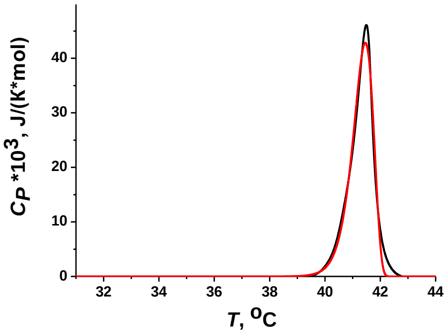
CP *103, J/(K*mol)
0
10
20
30
40
32
34
36
38
40
42
44
T, oC

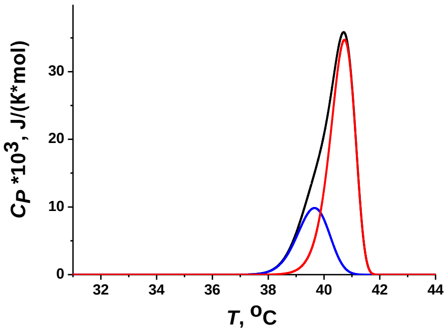
CP *103, J/(K*mol)
0
10
20
30
32
34
36
38
40
42
44
T, oC

3

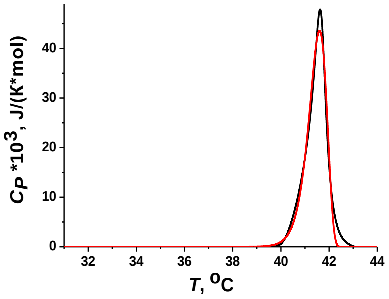
CP *103, J/(K*mol)
0
10
20
30
40
32
34
36
38
40
42
44
T, oC

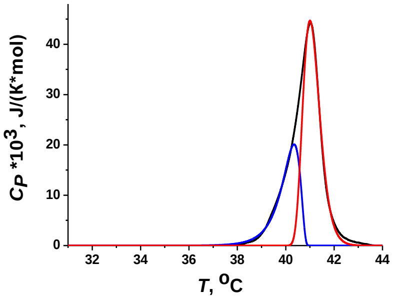
CP *103, J/(K*mol)
0
10
20
30
40
32
34
36
38
40
42
44
T, oC

4

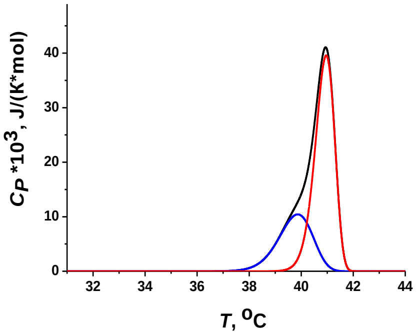
CP *103, J/(K*mol)
0
10
20
30
40
32
34
36
38
40
42
44
T, oC

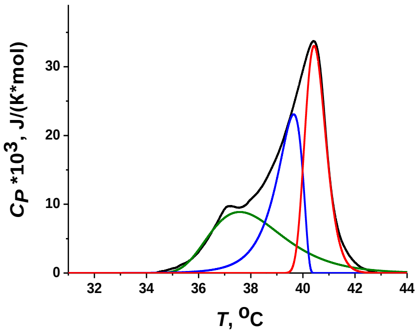
CP *103, J/(K*mol)
0
10
20
30
32
34
36
38
40
42
44
T, oC

5

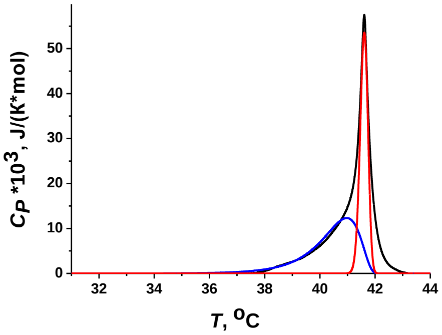
CP *103, J/(K*mol)
0
10
20
30
40
50
32
34
36
38
40
42
44
T, oC

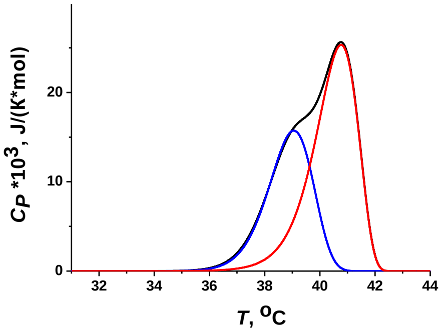
CP *103, J/(K*mol)
0
10
20
32
34
36
38
40
42
44
T, oC

6

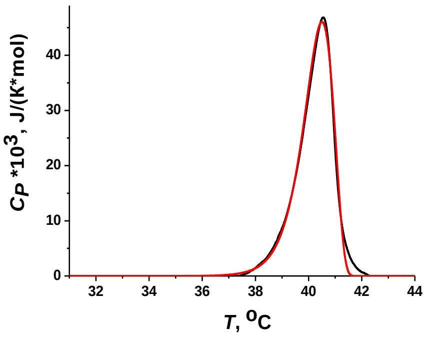

CP *103, J/(K*mol)
0
10
20
30
40
32 34 36 38 40 42 44
T, oC

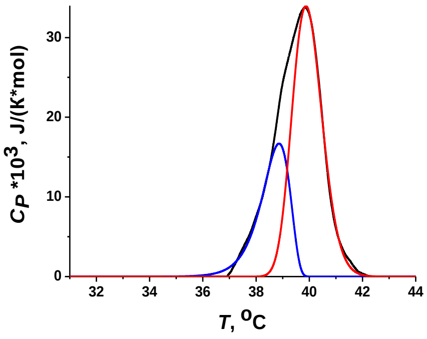

CP *103, J/(K*mol)
0
10
20
30
32 34 36 38 40 42 44
T, oC

7

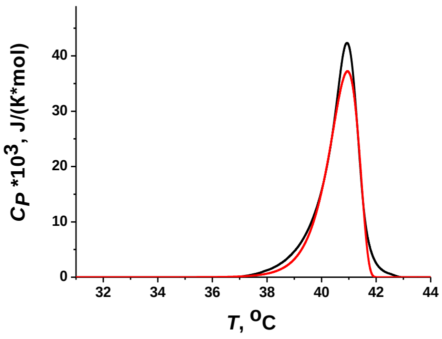

CP *103, J/(K*mol)
0
10
20
30
40
32 34 36 38 40 42 44
T, oC

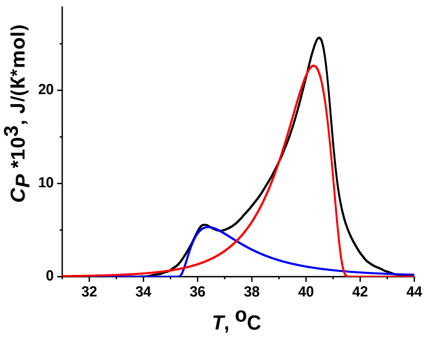

CP *103, J/(K*mol)
0
10
20
32 34 36 38 40 42 44
T, oC

8

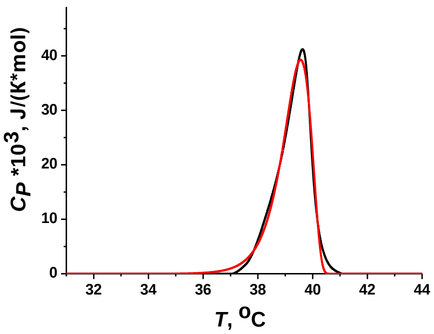

CP *103, J/(K*mol)
0
10
20
30
40
32 34 36 38 40 42 44
T, oC

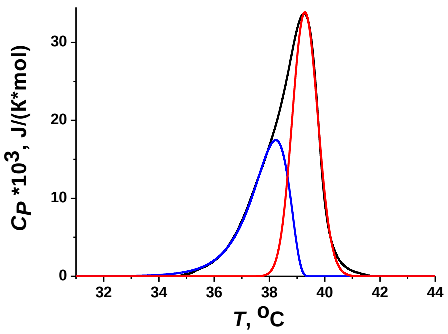

CP *103, J/(K*mol)
0
10
20
30
32 34 36 38 40 42 44
T, oC

9

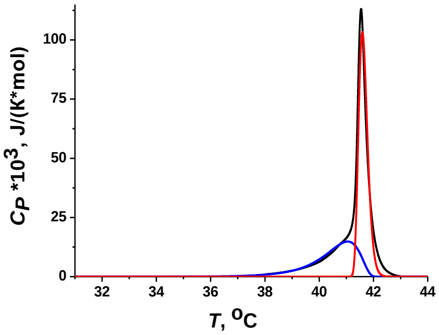

CP *103, J/(K*mol)
0
25
50
75
100
32 34 36 38 40 42 44
T, oC

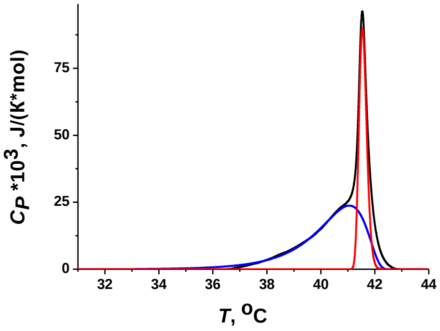

CP *103, J/(K*mol)
0
25
50
75
32 34 36 38 40 42 44
T, oC

10

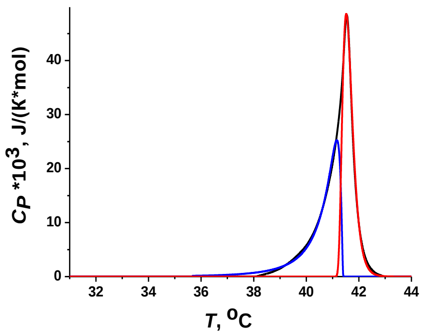
CP *10^3, J/(K*mol)
T, oC
0 10 20 30 40
32 34 36 38 40 42 44

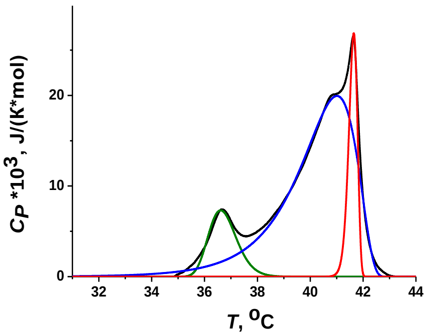
CP *10^3, J/(K*mol)
T, oC
0 10 20
32 34 36 38 40 42 44

11

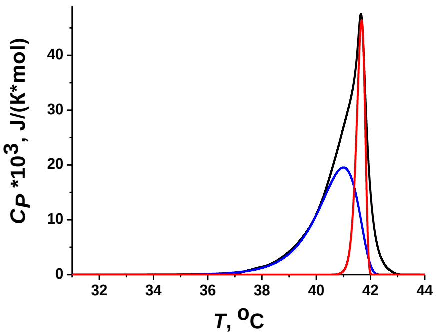
CP *10^3, J/(K*mol)
T, oC
0 10 20 30 40
32 34 36 38 40 42 44

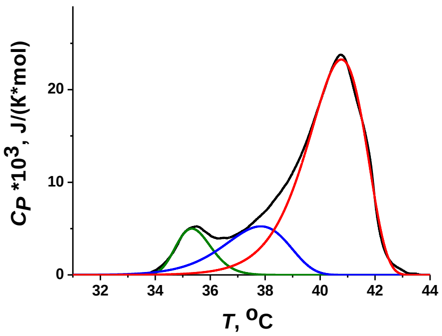
CP *10^3, J/(K*mol)
T, oC
0 10 20
32 34 36 38 40 42 44

12

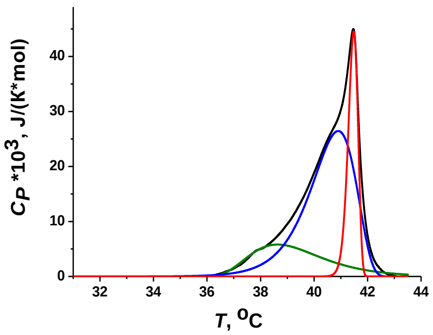
CP *10^3, J/(K*mol)
T, oC
0 10 20 30 40
32 34 36 38 40 42 44

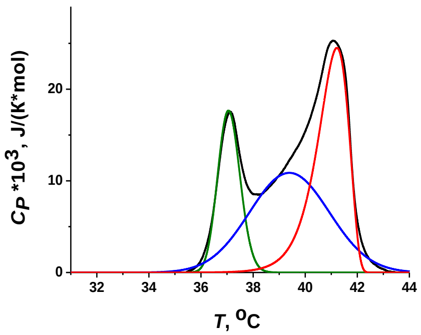
CP *10^3, J/(K*mol)
T, oC
0 10 20
32 34 36 38 40 42 44

Supplementary Figure S3. Reversibility of heating thermograms of DPPC liposomes in the absence (*black lines*) and presence of 100 (*red lines*) and 250 µg/ml (*green lines*) of chromone-containing allylmorpholines. Data of heating steps of two repetitive scans are presented.

agent	heating 1	heating 2
1	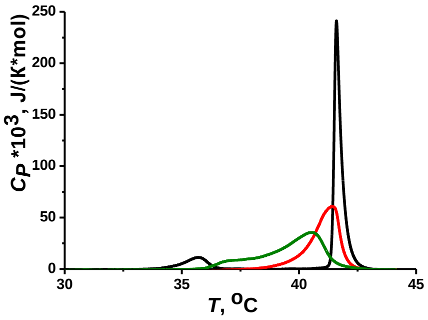	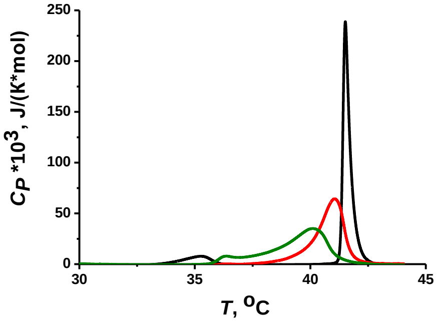
7	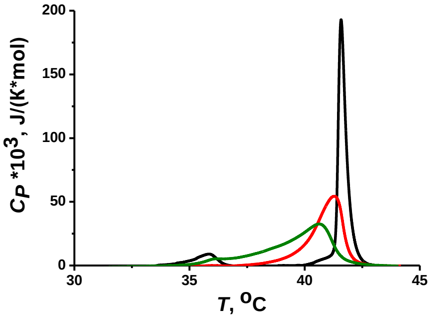	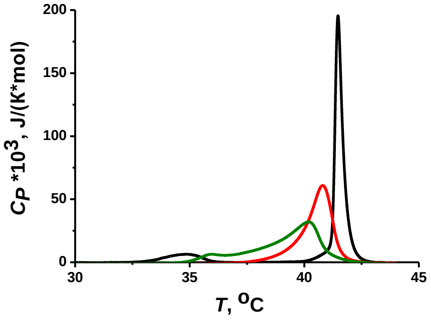
9	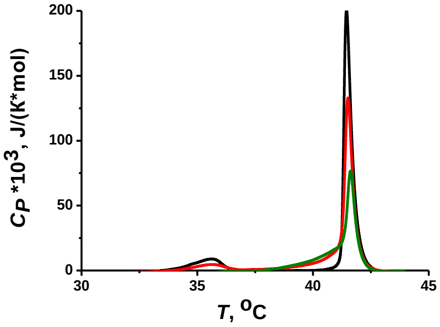	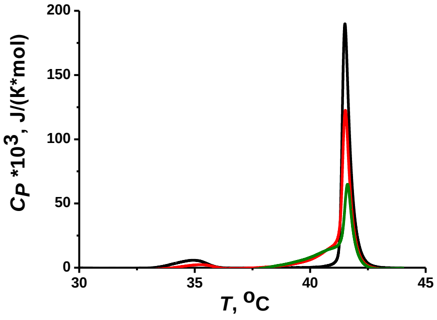

Supplementary Figure S4. The thermograms of lamellar-to-inverted hexagonal phase transition of POPE in the absence (*black line*) and presence derivative **7** at 100 µg/ml (*red line*).

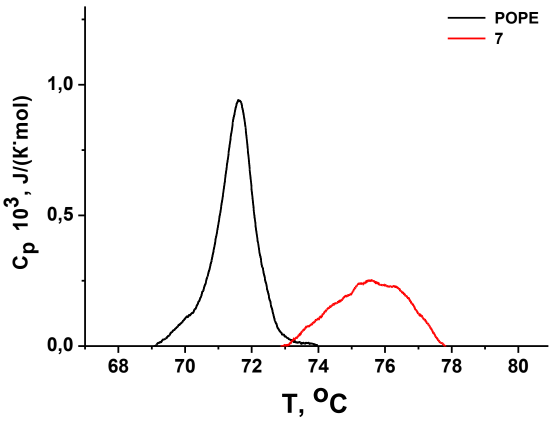

Supplementary Figure S5. The effects of chromone-containing allylmorpholines (**1**, **2**, **3**, **5**, **9**, **10**, **11**, and **12**) on the steady-state transmembrane current flowing through membranes modified by one-side addition of AmB. The moments of the addition of 100 µg/ml of **1**, **2**, **3**, **5**, **9**, **10**, **11**, and **12** to the bilayer bathing solution are indicated by arrows. The lipid bilayers were composed of POPC/CHOL (80/20 mol%) and bathed in 2.0 M KCl, pH 7.4. V = 50 mV.

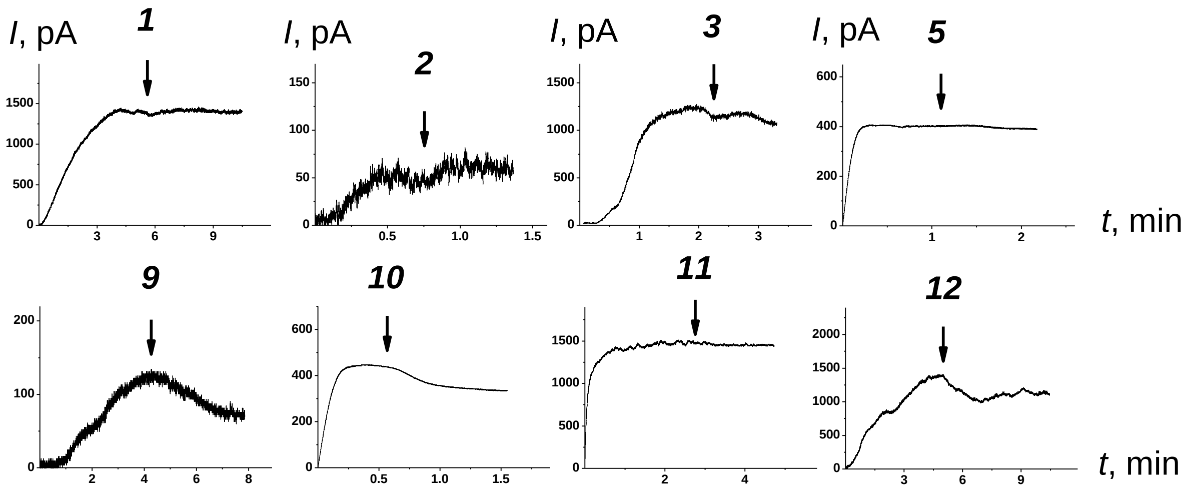

Supplementary Figure S6. The effects of allymorpholines ***1*** (**A**) and ***7*** (**B**) on lipid bilayers composed of POPC and bathed in 0.1 M KCl pH 7.4. The transmembrane voltage was 100 mV. Arrows indicate the moments of addition of chromone-containing allymorpholines to the membrane bathing solution.

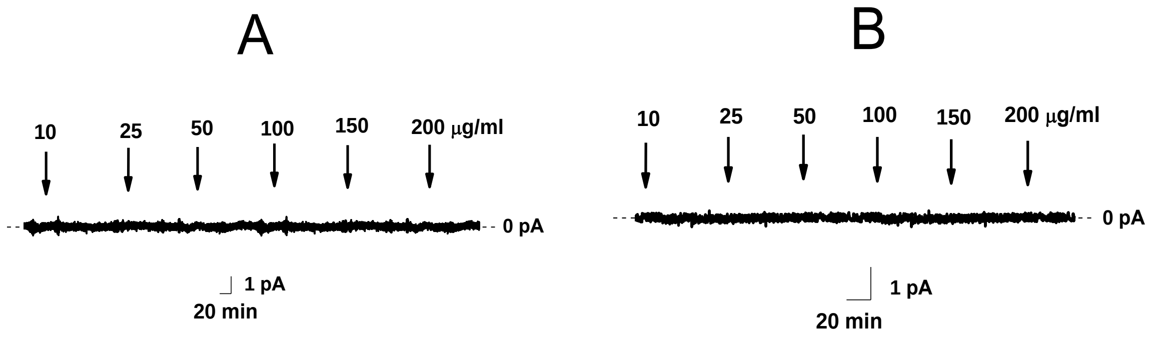

1.2 Supplementary Tables

Supplementary Table S1. The characteristics of tested chromone-containing allylmorpholine molecules.

agent	***number*** in (Chernov et al., 2018)	***molecular weight***	***pKa***[#]	***logP***[#]	***μ***[#], D
1	9e	428.75	6.21	3.53	4.21
2	9b	349.85	6.21	2.52	3.98
3	9f	367.84	6.21	2.81	4.56
4	9j	442.77	6.21	3.15	2.82
5	33b	428.84	6.42	4.90	2.94
6	9g	394.85	6.21	2.70	8.87
7	9a	384.3	6.21	3.07	4.39
8	25a	370.27	6.30	2.72	5.62
9	34a	468.46	6.27	6.14	4.73
10	33a	424.36	6.42	4.55	4.71
11	30a	412.35	6.28	4.32	4.29
12	31a	412.35	6.28	4.13	4.55

the values of *pKa* (the ionization constants), *LogP* (logarithms of the octanol/water partition coefficients), and *μ* (dipole moments) are predicted by MolGpKa, ACD/ChemSketch, and ORCA Software at PM6 level respectively (ACD/ChemSketch http://www.acdlabs.com).

Supplementary Table S2. The main peak decomposition/deconvolution analysis in the presence of chromone-containing allylmorpholines.

agent	***peak***	T_{m_i}, °C		$\frac{\Delta H_i/\Delta H_{cal}}{\sum_i \Delta H_i/\Delta H_{cal}}$, %	
		100 µg/ml	*250 µg/ml*	*100 µg/ml*	*250 µg/ml*
1	№1	41.5	40.3	100	57
	№2	–	39.3	–	32
	№3	–	36.5	–	11
2	№1	41.5	40.7	100	74
	№2	–	39.7	–	26
3	№1	41.5	41.0	100	78
	№2	–	40.5	–	22
4	№1	40.9	40.4	79	53
	№2	39.8	39.6	21	33
	№3	–	37.2	–	14
5	№1	41.5	40.8	76	71
	№2	40.9	38.9	24	29
6	№1	40.5	39.9	100	72
	№2	–	38.8	–	28
7	№1	41.0	40.2	100	84
	№2	–	36.2	–	16
8	№1	39.6	39.3	100	66
	№2	–	38.2	–	34
9	№1	41.5	41.5	86	82
	№2	41.0	41.0	14	18
10	№1	41.5	41.5	66	48
	№2	41.1	41.1	34	38
	№3	–	36.7	–	14
11	№1	41.5	40.7	59	72
	№2	41.3	37.6	41	14
	№3	–	35.2	–	14
12	№1	41.5	41.2	75	47
	№2	40.8	39.3	20	22
	№3	38.4	37.1	5	31

T_m of pure DPPC is equal to 41.5 ºC

Supplementary Table S3. T_m-hysteresis of DPPC in the presence of chromone-containing allylmorpholines[#].

	ΔT_h, °C	
agent	*100 μg/ml*	*250 μg/ml*
1	0.5 ± 0.1	0.8 ± 0.1
2	0.5 ± 0.1	0.8 ± 0.1
3	0.4 ± 0.1	0.9 ± 0.2
4	0.6 ± 0.2	0.8 ± 0.2
5	0.5 ± 0.1	1.2 ± 0.4
6	0.7 ± 0.2	0.8 ± 0.1
7	0.7 ± 0.1	0.9 ± 0.2
8	0.7 ± 0.1	0.9 ± 0.2
9	0.3 ± 0.1	0.5 ± 0.1
10	0.6 ± 0.1	0.9 ± 0.2
11	0.6 ± 0.1	0.9 ± 0.3
12	0.5 ± 0.1	1.2 ± 0.2

[#] ΔT_h of pure DPPC is equal to 0.4 ± 0.1.
